# Effects of Beetroot Juice on Recovery of Muscle Function and Performance between Bouts of Repeated Sprint Exercise

**DOI:** 10.3390/nu8080506

**Published:** 2016-08-18

**Authors:** Tom Clifford, Bram Berntzen, Gareth W. Davison, Daniel J. West, Glyn Howatson, Emma J. Stevenson

**Affiliations:** 1 Faculty of Health and Life Sciences, Department of Sport, Exercise & Rehabilitation, Northumbria University, Newcastle NE1 8ST, UK; tom.clifford@northumbria.ac.uk; 2 Faculty of Health Medicine and Life Sciences, Maastricht University, Maastricht 6211 LK, The Netherlands; bram.berntzen@student.maastrichtuniversity.nl; 3 Sport and Exercise Sciences Research Institute, Ulster University, Ulster BT52 1SA, Northern Ireland, UK; gw.davison@ulster.ac.uk; 4 Human Nutrition Research Centre, Institute of Cellular Medicine, Newcastle University, Newcastle NE2 4HH, UK; daniel.west@newcastle.ac.uk (D.J.W.); emma.stevenson@newcastle.ac.uk (E.J.S.); 5 Water Research Group, School of Environmental Sciences and Development, Northwest University, Potchefstroom 2520, South Africa

**Keywords:** beetroot juice, muscle damage, exercise recovery, repeated sprint exercise

## Abstract

This study examined the effects of beetroot juice (BTJ) on recovery between two repeated-sprint tests. In an independent groups design, 20 male, team-sports players were randomized to receive either BTJ or a placebo (PLA) (2 × 250 mL) for 3 days after an initial repeated sprint test (20 × 30 m; RST1) and after a second repeated sprint test (RST2), performed 72 h later. Maximal isometric voluntary contractions (MIVC), countermovement jumps (CMJ), reactive strength index (RI), pressure-pain threshold (PPT), creatine kinase (CK), C-reactive protein (hs-CRP), protein carbonyls (PC), lipid hydroperoxides (LOOH) and the ascorbyl free radical (A^•−^) were measured before, after, and at set times between RST1 and RST2. CMJ and RI recovered quicker in BTJ compared to PLA after RST1: at 72 h post, CMJ and RI were 7.6% and 13.8% higher in BTJ vs. PLA, respectively (*p* < 0.05). PPT was 10.4% higher in BTJ compared to PLA 24 h post RST2 (*p* = 0.012) but similar at other time points. No group differences were detected for mean and fastest sprint time or fatigue index. MIVC, or the biochemical markers measured (*p* > 0.05). BTJ reduced the decrement in CMJ and RI following and RST but had no effect on sprint performance or oxidative stress.

## 1. Introduction

Repeated sprint exercise (RSE), in which a number of short-duration maximal effort sprints (2–6 s) are completed intermittently with brief recovery periods (≤60 s), places a great deal of stress on the physiological and musculoskeletal systems [1]. The high energy turnover during RSE induces significant metabolic stress, triggering rapid perturbations in the nervous, immune, and endocrine systems [1], as well as an increased formation of reactive oxygen species (ROS) [2]. In addition, the high-force eccentric muscle contractions required to accelerate and decelerate during RSE places a great deal of mechanical stress on the musculoskeletal system, particularly the quadriceps and hamstring muscle groups [3]. It is therefore not surprising that team-sport players, who routinely engage in RSE in training sessions and matches, often display symptoms of muscle damage (i.e., muscle soreness and reduced muscle function) that can persist for several days [4,5,6]. Because the typical time between training sessions and or matches is often not sufficient for full recovery (≤72 h) athletes and coaches are continually seeking strategies that could help minimise the negative effects of muscle damage [7,8].

The exact mechanisms to explain the causes of muscle damage after RSE are not fully understood, but a host of factors such as muscle membrane damage, sarcomere disorganization, excitation-contraction coupling dysfunction, contractile protein degradation and inflammation are all likely to play a role [9,10]. Furthermore, it has been suggested that the generation of ROS in the days post-exercise, likely a consequence of inflammatory mediated repair processes, might exacerbate the existing muscle damage by degrading components of the cytosol that are integral to force production [11,12,13]. A number of studies have provided evidence of oxidative stress in the hours and days following RSE [2,6,14], suggesting that the endogenous antioxidant system is unable to cope with excess ROS production under these conditions. Thus, it would be reasonable to assume that the prolonged decrement in muscle function might be, at least in part, attributable to oxidative stress. This also makes the expectation tenable that interventions attempting to combat the excess production of ROS and control oxidative stress, such as antioxidants, could help accelerate the rate of muscle recovery following RSE.

While the nutritional antioxidants vitamin C and E have proven largely ineffective at attenuating muscle damage [15,16,17], there is growing support for the use of antioxidant-rich fruit and vegetable beverages as recovery aids [18]. Recently, we showed that supplementation with beetroot juice (BTJ) attenuated some aspects of muscle damage following high intensity plyometric exercise [19]. We proposed that one of the potential mechanisms by which of BTJ might have attenuated EIMD in this study was via its antioxidant effects. Although the antioxidant effects of BTJ has received little attention in the literature, findings from our previous work [20] and others [21,22] suggest that its antioxidant capacity is markedly higher than other vegetable juices, such as tomato and carrot juice, and also than several other drinks considered to have a high antioxidant capacity such as green tea, apple, cherry and cranberry juice [20,23,24]. The high antioxidant capacity of BTJ is due to the fact that several of the phytonutrient compounds it contains have been shown to scavenge ROS production in vitro and in vivo and subsequently limit cellular injury [25,26,27]. The most potent antioxidant molecules in BTJ are thought to be the betalain pigments, which are responsible for beetroot’s violet colour [28]. The betalains, and betanin in particular, are very effective electron donors that have been shown to not only attenuate ROS mediated injury but also to upregulate endogenous antioxidant enzymes and stimulate host defence [29,30,31,32]. In addition, BTJ is rich in nitrate, which, via its reduction to nitric oxide (NO) might have indirect antioxidant effects by supressing the accumulation of leukocytes [33], which are thought to be the main producers of ROS after muscle-damaging exercise [34]. Nonetheless, we failed to quantify oxidative stress in our aforementioned experiment [19] to confirm or refute this posit. Furthermore, the aforementioned findings were in recreationally active participants and therefore these results might not be directly transferable to better trained athletic populations.

To our knowledge, the effects of BTJ on muscle damage and recovery after activity incorporating RSE has not been investigated. Additionally, the effectiveness of such an intervention on subsequent performance has not been considered. Therefore, the main aim of this study was to examine whether BTJ can attenuate losses in muscle function and performance between two sport-specific repeated sprint tests (RST) performed 72 h apart. We also examined the effects of BTJ on biochemical markers associated with muscle damage, specifically oxidative stress, to try and discern what role (if any) the antioxidant potential of BTJ has on attenuating EIMD. Based on our previous findings [19], we hypothesized that: (1) BTJ would attenuate muscle function deficits and oxidative stress between and after the two repeated sprint tests; and (2) that performance during the second sprint test would be preserved with BTJ compared to a placebo.

## 2. Materials and Methods

### 2.1. Participants

Twenty male participants gave written informed consent for participation in this study (characteristics presented in Table 1). The sample size for this study was based on a priori power calculation. Based on a previous study [19], with a power of 0.80 and two tailed α level set at 0.05, the minimum number of participants required to detect an 8% difference in counter movement jump (CMJ) performance between groups (SD: 6%) was estimated as 10 per group. We selected CMJ as our primary outcome measure because it is believed to be the most sensitive test for detecting reductions in neuromuscular function after RSE [35]. Our secondary outcomes included other markers of neuromuscular function, repeated sprint performance, muscle pain, and biochemical markers of inflammation, oxidative stress and muscle damage (specific details in relevant sections below). All participants were collegiate team-sports players, competing in either soccer (*n* = 10), rugby (*n* = 5), basketball (*n* = 2) hockey (*n* = 2) or handball (*n* = 1) on a regular basis; all testing was performed at the end of the competitive season (between March 2015 and June 2015). Participant’s eligibility was assessed with a health screening questionnaire. None had any known food allergies, were suffering from a musculoskeletal injury, or had previous history of renal, gastrointestinal or cardiovascular complications or any other contraindication to the study procedures. For the 48 h prior to and throughout data collection, participants were prohibited from consuming alcoholic beverages, and instructed to avoid any strenuous exercise outside of the trial requirements. The study protocol received ethical approval from the Faculty of Health and Life Sciences ethics committee at Northumbria University. Approval was granted on the 26 February 2015 and assigned the following project identification code: HLSTC200115.

### 2.2. Experimental Design

**Figure 1:**
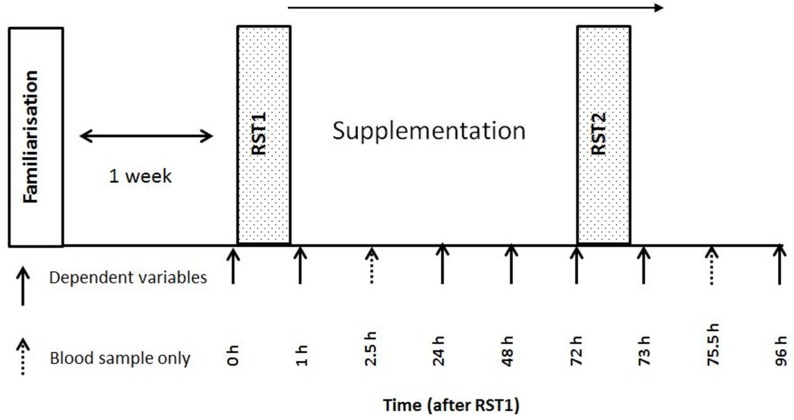
Schematic outline of study procedures.

This study employed a double-blind, placebo controlled, independent groups design. Participants were required to attend the laboratory for 6 visits over a 2 week period. The first visit was to familiarise the participants with the study procedures and randomly allocate them to either a beetroot juice (BTJ) or an isocaloric placebo (PLA) group. Their baseline maximal isometric voluntary contraction (MIVC) was used to match the groups. The principal investigator was responsible for the randomizing procedures. The next five visits were performed on consecutive days in the same laboratory at the same time of day and were preceded by an overnight fast. For the main trials, participants performed two repeated sprint tests separated by 72 h (visit 2 = RST1 and visit 5 = RST2) (see Figure 1 for schematic outline). A range of dependent variables were taken pre, 30-min post, 24, 48 and 72 h after RST1, and 30-min post and 24 h after RST2 to monitor recovery. On each occasion, dependent variables were performed in the following order: pressure-pain threshold (PPT), venous blood draw, CMJ, reactive strength index (RI) and MIVC. After completing the post-exercise measures participants consumed 1 serving of their allocated treatment, and returned to the lab 2.5 h post ingestion for a further blood sample. Another treatment was taken with an evening meal, and then at the same points (with breakfast and with an evening meal) for the following 3 days. All data collection took place in the exercise laboratories at Northumbria University.

### 2.3. Repeated Sprint Test

The RST consisted of 20 maximal-effort 30 m sprints, interspersed by 30 s of passive recovery. A 10 m deceleration zone was marked out at the end of each 30 m sprint, in which participants were required to stop within; the 30 s rest period commenced when participants had come to a halt. The RST was adapted from previous studies that showed repeated sprints with forced decelerations induce substantial muscle damage and fatigue in team-sport trained participants [3,36,37]. Furthermore, the muscle damage induced by an analogous RST seems to cause reductions in muscle function not different to those observed after intermittent sport simulations [38] and competitive matches [39]. Before performing each RST, participants undertook a standardized warm up as previously described [3]. Briefly, participants completed 400 m of self-selected jogging, a series of dynamic stretches, and sprints at 60% and 80% of maximal effort. Participants were then given a further 5 min to complete their own stretching. Timing gates (Brower Timing Systems, Draper, UT, USA) were positioned at 0 and 30 m to record sprint times. Participants were instructed to give maximal-effort for each sprint and were provided with strong verbal encouragement throughout. All testing took place in an air conditioned sprint track in similar environmental conditions.

### 2.4. Maximal Isometric Voluntary Contractions

MIVC of the right knee extensors was assessed as previously described [3,19]. Participants were seated and fitted to a portable strain gauge (MIE Medical Research Ltd., Leeds, UK) via a plinth placed just above the malleoli of the right ankle. In this positon, joint angle was adjusted to 90° of knee flexion using a goniometer and marked to ensure consistency across visits. Participants performed 3 maximal effort isometric contractions, each lasting 3 s, and separated by 60 s seated rest. The peak value in Newton’s (N) was used for analysis. Coefficient of variation (CV) for this protocol in our lab was calculated as 1.1%.

### 2.5. Counter Movement Jump

CMJ height was determined from flight time using an optical measurement system (Optojump next, Bolzano, Italy). Participants started the movement upright with hands fixed to their hips and after a verbal cue, descended into a squat prior to performing a maximal effort vertical jump. Participants performed 3 maximal efforts, separated by 30 s standing recovery. Mean height (cm) was used for analysis. The CV for this protocol in our lab was calculated as 2.1%.

### 2.6. Reactive Strength Index

Reactive strength index (RI) was used to measure the impact of muscle damaging exercise on participant’s ability to utilize the stretch shortening cycle and perform explosive actions. In a similar fashion to previous studies [40], participants performed a drop jump from a 30 cm box and, upon landing, immediately jumped vertically, with instructions to minimise ground contact time while maximising jump height. RI was calculated as jump height divided by ground contact time (cm/ms) recorded from an optical measurement system (Optojump next). Participants performed 3 maximal efforts separated by 30 s of passive (standing recovery) with the mean height of the 3 jumps used for analysis. The CV for this protocol was calculated as 1.9% in our lab.

### 2.7. Treatments and Dietary Control

Participants consumed 2 bottles (250 mL per bottle) of their assigned treatment (BTJ or PLA) on the day, 24, and 48 h after RST1 and 30-min post RST2, equating to 8 servings in total. One bottle was consumed 30-min after each trial, and one with an evening meal. The BTJ was supplied by Gs Fresh Ltd., (Cambridgeshire, UK) and consisted of 99% beetroot juice concentrate, nitrate and other phytonutrients; specific details of the antioxidant capacity and phytonutrient content of this drink can be found elsewhere [20]. The PLA consisted of a low fruit containing (<1%) squash (Kia Ora, Coca Cola Enterprises, Uxbridge, UK), flavourless protein powder (Arla Foods, Amba, Denmark) and maltodextrin powder (Myprotein, Manchester, UK) providing a negligible amount of phytochemicals and nitrate. Treatments were closely matched for volume, macro-nutrient and energy content, but differed in antioxidant capacity and nitrate content (see Table 2). Participants were provided with food dairies to record their intake 24 h prior to RST1 up until data collection was complete (24 h post RST2; 5 days in total). Average energy and macronutrient intake for each group is presented in Table 3. To comply with the double-blind, randomized design, drinks were provided in identically masked bottles, only distinguished by a single letter code. These were prepared by an individual not involved in data collection. As detailed in a previous study [19], due to the distinct taste of BTJ, the PLA was not matched for taste and texture, only energy content. While others have used nitrate depleted BTJ as a PLA so that the taste is the same, this is not a true PLA because it will still contain many other bioactive constituents (i.e., phenolics and betalains) that, as outlined in the introduction, could favourably affect recovery. Thus, this would not have been plausible in the present study. Rather, in an attempt to overcome this, the participants were not informed of what the specific drinks being investigated were. The only information they received was that they were antioxidant-containing drinks used for recovery. This ensured that the participants did not know the overall aim of the study, eliminating any bias based on pre-conceptions regarding BTJs potential ergogenic effects. Additionally, because we employed an independent groups design, participants were never aware of the taste/texture of the other treatment under investigation.

### 2.8. Muscle Soreness

Site specific muscle soreness was assessed with a handheld algometer (Wagner Instruments, Greenwich, CT, USA). A cylindrical flat headed pad (1 cm diameter) was applied with increasing pressure on the muscle belly at three pre-marked sites: vastus lateralis, mid-way between the superior aspect of the greater trochanter and head of the tibia, rectus femoris, mid-way between the anterior patella and inguinal fold, and gastrocnemius, most medial aspect of the calf at relaxed maximum girth. The point at which the participant signified they felt pain was recorded in N^2^ as pressure pain threshold (PPT). Sites were re-marked on each visit to ensure consistency between recordings. The average of two values from each site was used for analysis, unless the difference between the two values was >10 N^2^ apart, in which case a third recording was taken, and the average of the two closest values used for analysis.

### 2.9. Blood Sampling

Venous blood was obtained via venepuncture from a branch of the basilica vein at the antecubital fossa. Samples were collected into di-potassium ethylene diamine tetra-acetic acid (EDTA) (1 × 10 mL) and serum vacutainers (1 × 10 mL). EDTA tubes were immediately centrifuged at 3000× *g* (4°) for 10 min, while serum tubes were allowed to clot for 45 min before centrifugation. Plasma and serum supernatant was aspirated into a series of aliquots and stored at −80 °C for later analysis.

### 2.10. Biochemical Analysis

High sensitivity C-reactive protein (hs-CRP) and creatine kinase (CK) were measured in serum using an automated system based on an electrochemiluminescence method (Roche Modular, Roche Diagnostics, Indianapolis, IN, USA. The typical CV for this method is <2%. Plasma protein carbonyls were measured using a commercially available assay kit (Cayman Chemical, Ann Arbor, MI, USA). Lipid hyroperoxides (LOOH) were measured in serum using the ferrous iron/xylenol orange (FOX) assay (Wolff 1994). The FOX assay determines the susceptibility to iron-induced LOOH formation in blood; consequently, the presence of iron ions in the assay protocol might lead to slightly higher LOOH values compared with other methods. Absorbance was read at 560 nm using a spectrophotometer (U-2001, Hitachi, Berkshire, UK) (range 0–5 μmol·L^−1^).

Ascorbyl free radical determination was quantified at room temperature using a Bruker EMX series X-band EPR spectrometer (Bruker, Karlsruhe, Germany). 1 mL of plasma was mixed thoroughly with 1 mL of dimethyl sulfoxide (DMSO) and slowly flushed into an aqua X multiple bore cavity cell. The EMX parameter settings were frequency, 9.785 GHz; microwave power, 20 mW; modulation frequency, 100 kHz and modulation amplitude, 1.194 G. All EPR spectra were subjected to 3 scans identically filtered and analysed using WinEPR software (Version 3.2, Bruker WinEPR, Coventry, UK). The average spectral peak-to-trough line amplitude was used to determine free radical concentration.

### 2.11. Data Analysis

All data are expressed as mean ± standard deviation (SD) and were analysed using IBM SPSS Statistics 22 for Windows (Surrey, UK). Participant’s food diaries (5 days) were analysed for macronutrient content using dietary analysis software (Nutritics LTD, Dublin, Ireland). Differences between participant group characteristics were analysed with an independent samples *t*-test. CMJ, RI, MIVC and PPT were measured using a mixed model ANOVA; 2 group levels (BTJ vs. PLA) by 7 time levels (pre, post, 24, 48, 72, 73 and 96 h post RST1). The same ANOVA was use to analyse all blood indices but with 2 additional time levels (2.5 h post RST1 and 2.5 h post RS2). A separate ANOVA was used to measure for differences between RST1 and RST2; 2 group levels (BTJ vs. PLA) by 2 time levels (pre and post). In the event of a significant interaction effect (group * time) Fisher LSD *post hoc* analysis was performed to locate where the significant differences occurred. Statistical significance was set at *p* < 0.05 prior to analyses. To estimate the magnitude of the supplements effects, Cohen’s *d* effect sizes (ES) were calculated with the magnitude of effects considered either small (0.20–0.49), medium (0.50–0.79) and large (>0.80).

## 3. Results

There were no between group differences in age, height, mass or baseline MIVC strength (Table 1; *p* > 0.05), indicating that the groups were well matched prior to testing. Furthermore, there were no differences in participant’s energy and macronutrient intake 24 h prior to and throughout the duration of the study (Table 3; *p* > 0.05). No adverse effects were reported by the participants throughout the trial.

### 3.1. Repeated Sprints

RPE showed no bout (*p* = 0.925) or interaction effects (*p* = 0.584) between RST1 and RST2, indicating that perceived exertion was not different for both bouts (Table 4). This was reflected in the sprint data, as fastest sprint time and fatigue index were not different between repeated sprint bouts, showing no main effects of time, bout, or bout * group interactions (*p* > 0.05). No group or group * bout interaction effects were present (*p* > 0.05).

### 3.2. Functional Measures

**Figure 2:**
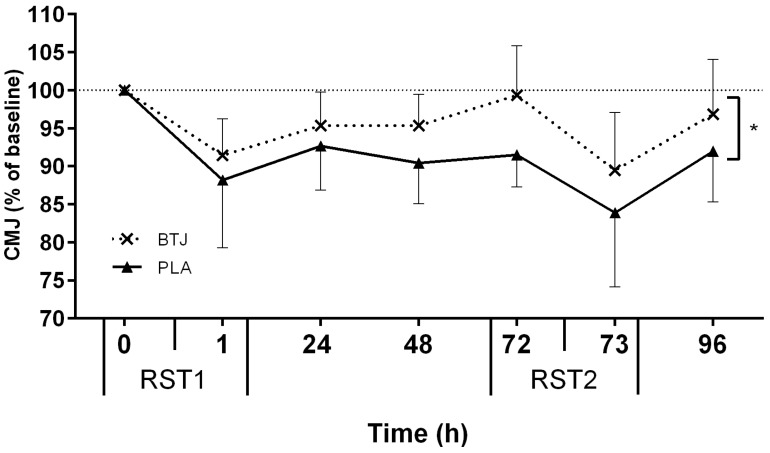
Percentage changes in counter movement jump (CMJ) height between repeated sprint tests (RST1 and RST2). * Represents group difference (beetroot juice (BTJ) vs. placebo (PLA); *p* < 0.05). Values are mean ± SD (*n* = 10 per group).

**Figure 3:**
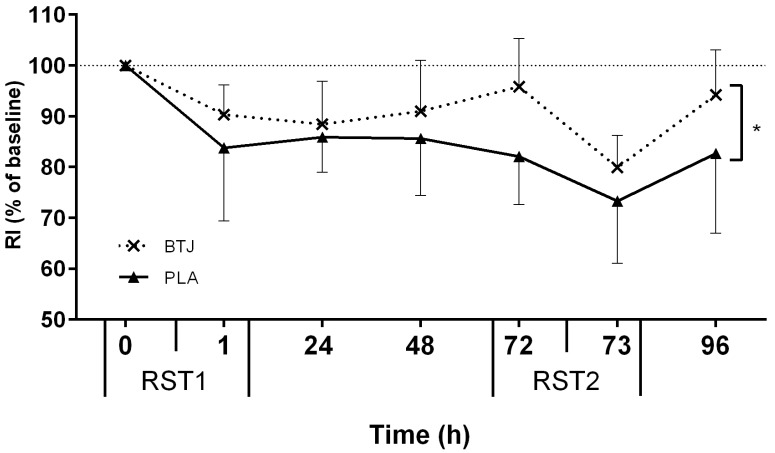
Percentage changes in reactive strength index (RI) between repeated sprint tests (RST1 and RST2). * Represents group difference (beetroot juice (BTJ) vs. placebo (PLA); *p* < 0.05). Values are mean ± SD (*n* = 10 per group).

**Figure 4:**
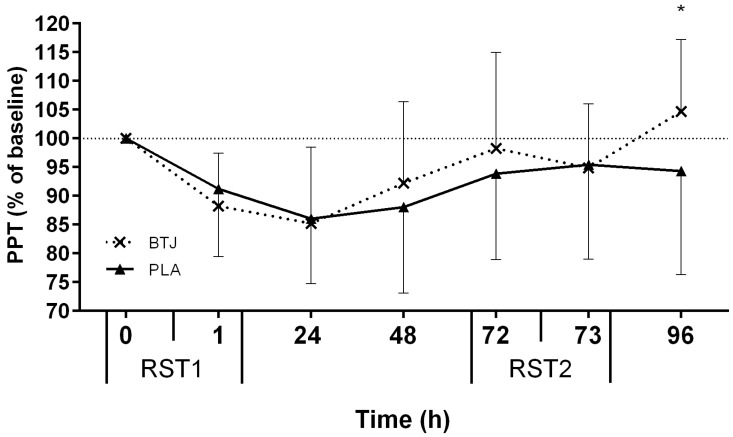
Percentage changes in pressure pain threshold (PPT) between repeated sprint tests (RST1 and RST2). Values presented are average of the three sites measured (calf (CF), rectus femoris (RF) and vastus laterialis (VL). * Indicates interaction effect (beetroot juice (BTJ) vs. placebo (PLA); *p* < 0.05). Values are mean ± SD (*n* = 10 per group).

All tests of neuromuscular function (CMJ, MIVC, RI), and PPT, showed main effects for time (*p* < 0.05), indicating that the RST induced muscle damage. Immediately post RST1, CMJ height was reduced by 11.8% ± 8.9% and 9.6% ± 4.8% (of baseline values) in the BTJ and PLA groups, respectively. A group effect showed that CMJ height appeared to recover quicker in BTJ vs. PLA throughout the remainder of the testing period (*p* = 0.048; Figure 2). Although no group*time interaction effects were present (*p* = 0.176), there was a large effect size (1.86) at 72 h post RST1 whereby CMJ height in the BTJ group was 7.6% higher than the PLA group. A group effect for RI (*p* = 0.030) showed that the maintenance of RI performance was also greater in BTJ vs. PLA throughout the trial (Figure 3). As with CMJ, a large effect size (1.43) was evident at 72 h post RST1 where RI had returned to 95.8% ± 9.5% of baseline values in BTJ compared to 82% ± 9.5% in PLA. There were no group effects for PPT (*p* = 0.368); however, an interaction effect was observed (*p* = 0.013; Figure 4). *Post-hoc* analysis revealed a group difference at 96 h post RST1 (*p* = 0.012; ES = 0.57); in the BTJ group, PPT had recovered to 104.7% ± 12.5% of baseline values, while in the PLA group, PPT was 94.3% ± 18% of baseline values. There were no significant group or interaction effects for MIVC (*p* > 0.05).

### 3.3. Biochemical Indices

Serum concentrations of hs-CRP remained close to baseline values throughout the trial, showing no time, group or interaction effects (*p* > 0.05). Serum CK showed main effects for time (*p* < 0.001), with the greatest increases observed 2.5 and 24 h post RST1 and RST2 in both groups (*p* > 0.05; Table 5). However, no group of interaction effects were observed (*p* > 0.05). A main effect for time was observed for serum LOOH (*p* < 0.001); LOOH was elevated immediately and at 2.5 h post-RST1 in both groups before returning to baseline 24 h post. A transient increase in LOOH was also evident at 75 h (2.5 h post RST2) but by 96 h had recovered to pre-exercise values. No group or interaction effects were present for LOOH (*p* > 0.05). Protein carbonyls, a common measure of protein oxidation, remained largely unchanged after both sprint bouts and showed no group or interaction effects (*p* > 0.05). Likewise, A^•−^, as measured by EPR, showed no time, group or interaction effects throughout the trial (*p* > 0.05).

## 4. Discussion

The main finding of the present study was that beetroot juice, when compared to a placebo, was able to accelerate the recovery of CMJ and RI performance and reduce pain after a muscle-damaging RST, but had no influence on sprint performance. Markers of systemic oxidative stress or other biochemical indices associated with muscle damage were unaffected by beetroot juice supplementation.

In both the BTJ and PLA groups CMJ and RI significantly decreased after RST1, indicating the presence of muscle damage; however, both CMJ and RI recovered quicker with BTJ (vs. PLA) during the following +96 h (Figure 2 and Figure 3, respectively). This was most evident at 72 h after the first sprint bout (RST1), whereby CMJ and RI were still significantly lower than baseline values, but restored close to pre-exercise values in BTJ group. These findings are consistent with our previous work, in which we reported 3 days of BTJ supplementation enhanced the recovery of CMJ performance 72 h after plyometric activity [19].

Interestingly, although BTJ appeared to enhance the recovery of the dynamic muscle function (CMJ and RI), isometric strength (MIVC) was unaffected by BTJ supplementation. As previously suggested [19], perhaps this discrepancy can be explained by the different movement patterns (i.e., static vs. dynamic function) and specific abilities each test measures (power vs. isometric strength). Both CMJ and RI are arguably more ecologically valid tests of functional recovery than MIVC, particularly for team-sports players as their movement patterns more closely reflect the activity required for performance [35]. We initially hypothesized that sprint performance would be reduced in RST2 compared to RST1; a consequence of muscle damage, and that this reduction would be attenuated with BTJ supplementation. However, contrary to our hypothesis, aside from a non-significant decrease in average sprint time (Table 3), sprint performance was largely unaffected in RST2 compared to RST1. The reductions in muscle function were not different ≤24 h after both sprints tests though, suggesting that the participants did not become accustomed to the sprint test after the first bout. Additionally, BTJ had no influence on any aspect of sprint performance, although perhaps our ability to detect any differences between groups was limited by the lack of change in performance between the two sprint tests. Nonetheless, the fact that sprint performance was unchanged seems to contrast with other studies who reported sprint times to still be slower than pre-exercise values up to 72 h after an RST similar to the present study [36,41,42]. Because the muscle-damaging RST was fairly similar between these studies (in fact, the one in the present study was designed to be more challenging), perhaps the divergent findings between these studies and the present one is due, in large part, to the different training status of the participants. The participants in the present study were experienced team-sports players who regularly perform RSE as part of training and matches and, thus, may have been less vulnerable to prolonged decrements in sprint performance than recreationally active participants tested in some of the other studies [41,42].

In addition, the fact that CMJ and RI were still significantly depressed at 72 h post RST1, but sprint performance was not, suggests that there is dissociation between these tests of dynamic muscle function (CMJ and RI) and repeated sprint ability. Indeed, previous literature appears to be equivocal on how well sprint and jump tests correlate. Some studies demonstrate that the time course of recovery for CMJ and sprint performance are not different after muscle-damaging RSE [36,41,42], while others agree with the present study [35,39,43], and have found that reductions in CMJ are more prolonged than sprint decrements. A recent study attempted to address this issue by comparing CMJ, drop jumps (DJ) and a 20 m sprint test after intermittent exercise and concluded that sprint performance seemed to recover more rapidly than both CMJ and DJ performance, both of which were still below pre-exercise values 72 h post-exercise [35]. This led the authors to suggest that CMJ and DJ are more sensitive tests of prolonged changes in neuromuscular function, which could provide and explanation for the dissociation between the jump and sprint tests in the present study.

Due to the fact that oxidative stress has been associated with muscle damage after eccentric-heavy exercise [14,44], and that beetroot and its constituents have been shown to act as antioxidants [25,29], we hypothesized that BTJ could attenuate muscle damage by protecting cells against oxidative stress. However, our findings do not support this contention. We found no evidence that BTJ attenuated oxidative stress as both indirect markers (LOOH and PC) and a direct marker of free radical production (A^•−^) were not different between the BTJ and PLA groups at all-time points (Table 5). These data are in contrast to a number of previous studies that found antioxidant-rich food supplements reduced oxidative stress after high intensity sprint exercise [14,45,46]. However, unlike these studies, we did not find any evidence of oxidative stress throughout the trial, apart from an increase in LOOH immediately post and 2.5 h post both sprint tests (Table 5). The modest increase in these markers was unexpected, as previous studies reported large systemic elevations in oxidative stress up to 48 h after high intensity intermittent cycling exercise [2,14] an activity which, in comparison to running, typically results in less oxidative stress because of the absence of an extensive eccentric component [34]. The divergent findings in oxidative stress response between the present and aforementioned studies could, therefore, be explained by the different biochemical markers examined and/or analytical techniques used. Jowko and colleagues [14] for instance, noted systemic increases in total antioxidant capacity (TAC), superoxide dismutase and glutathione peroxidase (GPX) 24 h after exercise and Bogdanis and colleagues [2] noted increases in TAC, PC and GPX; thus, neither study measured LOOH or A^•−^ formation, as in the present study. Although this study and [2] both measured PC, different analytical methods were used, which could account for the discrepant results. Nonetheless, EPR spectroscopy is considered a valid and sensitive method for direct detection of excessive free radical production [47,48], and the fact that we found no evidence of an increase in our data perhaps draws into question the reliability of the indirect biomarkers in other studies using a similar protocol.

The fact that muscle damage was clearly evident in the days after both RST tests but oxidative stress was not, suggests that muscle damage occurred independent of any systemic changes in oxidative stress. This would perhaps suggest that ROS have a limited role in the muscle damage process post-exercise. However, it cannot be ruled out that oxidative stress occurred, but was confined predominately to muscle cells and surrounding tissues. Unfortunately, we did not measure muscle samples in our study, and as such, this supposition is speculative. A recent review however, concluded that skeletal muscle is a prime producer of ROS following exercise; so, intuitively, oxidative stress would be expected to be greater in muscle than perhaps the circulation [49]. We recognize that the inability to obtain muscle biopsy samples for oxidative stress measures could be considered a limitation of this study. Alternatively, the muscle damage we observed could have been unrelated to oxidative stress. Instead, the muscle damage could have been caused by other biochemical changes within muscle, such as increased inflammation and calpain activity [50,51] or damage to components involved in the excitation-contraction coupling pathway, as previously suggested [52].

Serum CK concentrations, incorporated as a surrogate marker of sarcolemma damage, were not different in both groups after exercise (Table 5). The increase in CK after the RST was similar to previous reports [3,42], as was the lack of a suppressive effect with an antioxidant-rich food beverage [19,23,45]. These data suggest that improved sarcolemma integrity cannot explain the enhanced rate of recovery by BTJ in this study.

Because we found no changes in oxidative stress between groups, the beneficial effects of BTJ on the recovery of CMJ and RI cannot be attributed to an antioxidant effect of the juice. This suggests that mechanisms other than antioxidant effects were possibly involved. It was beyond the scope of this study to examine the role of other mechanisms by which BTJ could attenuate muscle damage, but owing to the seemingly pleotropic nature of phenolic and betalainic compounds and NO, there are a number of possible candidates. For instance, other effects associated with phenolic compounds and NO donors akin to BTJ are anti-inflammatory [25,33] and regenerative, in so far as they appear to have a regulatory role in phagocytosis and promote satellite cell proliferation in skeletal muscle [53,54,55]. Increasing in vivo NO availability has also demonstrated additional biochemical effects that, conceivably, could contribute to improved functional recovery after exercise, such as reduced calpain activity [56], increased muscle blood flow [57,58], and enhanced muscle power potential, possibly via improved Ca^2+^ handing [59,60]. Thus, there are a number of potential mechanisms that could explain why BTJ supplementation was able to enhance the recovery of muscle function, independent of antioxidant effects. However, since none of these mechanisms were measured *per se*, we can only speculate the role, if any, that they may have had in the present study’s findings. The potential role and their relative contributory effects require further exploration.

Participants in the BTJ group reported a significantly higher PPT than the PLA group 24 h after the second sprint test (Figure 4). Reduced muscle pain when antioxidant-rich food supplements are taken after muscle-damaging exercise has been reported by our group [19] and others [61]. The mechanism by which BTJ might attenuate muscle pain is unclear however. Previous reports suggest that the betalains in beetroot are responsible for its analgesic effects, most likely via an anti-inflammatory related mechanism [26,62]. The possibility that an anti-inflammatory mechanism would be involved is supported by data that suggests muscle pain after exercise may stem from the release of inflammatory and noxious stimuli (i.e., bradykin and nerve growth factor) due to tears at the extracellular matrix [63,64]. Perhaps BTJ acts to dampen inflammatory responses or desensitize pain receptors, as has been suggested with ginger [65] and curcumin supplements [66]; however, whatever the precise mechanisms, they are likely to occur at the skeletal muscle level.

It is also unclear why BTJ only improved PPT 24 h after RST2 in the present study and not at earlier time points, as was shown in our earlier work [19]. A previous study did observe greater reductions in pain scores after participants took betalain-rich beetroot supplements for 5–10 days compared to 1 day [26], which, coupled with our data, suggests that the analgesic effects of BTJ might be augmented with longer-term dosage regimens. Such a possibility needs to be investigated in future studies.

In conclusion, this study demonstrates that consuming BTJ for 4 days after a muscle damaging RST attenuated muscle pain and decrements in dynamic muscle function, as measured by CMJ and RI. These effects did not translate to improved recovery of isometric strength or sprint performance however. These data suggest BTJ could be applied as a post-exercise recovery strategy to attenuate losses in some aspects of dynamic muscle function in team-sports players between bouts of repeated sprint exercise; however, because sprint performance was unchanged, how transferable these findings are to real-world team-sport competition is unclear. Future studies are needed to clarify the underlying cellular mechanisms, as the beneficial effects of BTJ were shown to be unrelated to systemic changes in oxidative stress or other biochemical markers of muscle damage.

## Figures and Tables

**Table 1:** Descriptive data for participants in the beetroot juice (BTJ) and placebo (PLA) groups.

Group	Age (Years)	Height (m)	Mass (kg)
BTJ	23 ± 3	1.83 ± 0.90	76.8 ± 9.5
PLA	21 ± 2	1.77 ± 0.51	73.4 ± 12.4

Values are mean ± SD (*n* = 10 per group). No significant differences were detected between groups for any variable (*p* > 0.05).

**Table 2:** Energy and macronutrient content, trolox equivalence antioxidant capacity (TEAC) and nitrate content of the beetroot juice (BTJ) and placebo (PLA) supplements.

Treatment	BTJ	PLA
Energy (Kcals)	81	77
Volume (mL)	250	250
Carbohydrate (g)	16.4	16.4
Protein (g)	2.8	2.8
Fat (g)	0.4	Trace
Nitrate (mg)	≥143	Trace
TEAC * (mmol∙L^−1^)	11.4 ± 0.2	0.25 ± 0.02

* Estimation based on previous analyses [20].

**Table 3:** Average intake and macro nutrient composition of participants diets (average of 5 days).

	Mean Dietary Intake (5 Days)	
	BTJ	PLA
Energy (Kcal)	2554 ± 682	2448 ± 390
Carbohydrates (%)	41 ± 5	43 ± 6
Protein (%)	21 ± 3	24 ± 7
Fat (%)	38 ± 7	33 ± 9

Values are mean ± SD (*n* = 10 per group). No significant differences were detected between groups for any variable (*p* > 0.05).

**Table 4:** Sprint and RPE data for the beetroot juice (BTJ) and placebo (PLA) groups in the first and second repeated sprint tests (RST1 and RST2, respectively).

Group	Average Sprint Time (s)	Fastest Sprint Time (s)	Fatigue Index (%)	RPE
**BTJ**				
RST1	4.65 ± 0.25	4.41 ± 0.23	5.60 ± 2.13	15 ± 1
RST2	4.66 ± 0.24	4.38 ± 0.17	6.48 ± 2.66	15 ± 1
**PLA**				
RST1	4.70 ± 0.15	4.48 ± 0.14	4.91 ± 1.51	14 ± 2
RST2	4.77 ± 0.20	4.53 ± 0.15	5.19 ± 3.21	14 ± 2

**Table 5:** Maximal isometric voluntary contractions (MIVC), high sensitivity-C-reactive protein (hs-CRP), creatine kinase (CK), lipid hydroperoxides (LOOH), protein carbonyls (PC) and plasma ascorbate free radical (A^•−^) for the beetroot juice (BTJ) and placebo (PLA) group’s pre-RST1 (0 h)—96 h post.

	0 h	1 h	2.5 h	24 h	48 h	72 h	73 h	75.5 h	96 h
MIVC (N) *									
**BTJ**	601 ± 89	554 ± 75		545 ± 92	558 ± 94	579 ± 121	516 ± 101		558 ± 75
**PLA**	590 ± 123	541 ± 128		535 ± 120	544 ± 130	540 ± 126	509 ± 124		537 ± 122
**hsCRP (mg∙L^−1^)**									
**BTJ**	0.6 ± 0.72	0.61 ± 0.75	0.57 ± 0.69	0.64 ± 0.56	0.64 ± 0.58	0.52 ± 0.52	0.52 ± 0.49	0.46 ± 0.50	0.48 ± 0.50
**PLA**	0.44 ± 0.39	0.46 ± 0.45	0.44 ± 0.42	0.52 ± 0.52	0.4 ± 0.25	0.34 ± 0.21	0.35 ± 0.21	0.38 ± 0.22	0.38 ± 0.24
CK (IU∙L^−1^) *									
**BTJ**	188 ± 62	219 ± 68	383 ± 197	542 ± 461	406 ± 252	310 ± 145	349 ± 163	474 ± 246	516 ± 210
**PLA**	318 ± 145	362 ± 154	518 ± 274	592 ± 321	435 ± 255	387 ± 273	433 ± 290	623 ± 423	749 ± 423
LOOH (mmol∙mL^−1^) *									
**BTJ**	1.49 ± 0.25	1.68 ± 0.23	1.69 ± 0.37	1.33 ± 0.36	1.46 ± 0.19	1.47 ± 0.17	1.53 ± 0.40	1.74 ± 0.31	1.44 ± 0.21
**PLA**	1.53 ± 0.14	1.79 ± 0.23	1.77 ± 0.40	1.54 ± 0.16	1.57 ± 0.14	1.53 ± 0.12	1.69 ± 0.23	1.94 ± 0.75	1.44 ± 0.14
**PC (μmol∙L^−1^)**									
**BTJ**	14 ± 6	16 ± 0	15 ± 6	17 ± 5	18 ± 7	14 ± 5	16 ± 5	17 ± 10	15 ± 6
**PLA**	16 ± 8	19 ± 6	14 ± 6	13 ± 5	14 ± 5	18 ± 9	16 ± 7	15 ± 5	15 ± 4
**A^•−^ (AU)**									
**BTJ**	5567 ± 1898	6111 ± 2145	5883 ± 2044	6247 ± 1846	6202 ± 1911	5809 ± 2238	5752 ± 1854	5422 ± 2002	5862 ± 1802
**PLA**	6372 ± 1454	6730 ± 1337	6559 ± 2027	5674 ± 1716	5225 ± 1088	6013 ± 671	5433 ± 1521	6354 ± 1315	5819 ± 975

AU = Arbitrary unit; * denotes time effect (*p* < 0.05).
